# Effects of separate and combined estradiol and progesterone administration on fear extinction in healthy pre-menopausal women

**DOI:** 10.1038/s41398-024-03079-4

**Published:** 2024-10-24

**Authors:** Michael Kaczmarczyk, Christian Eric Deuter, Hanna Deus, Anna Kallidou, Christian J. Merz, Julian Hellmann-Regen, Christian Otte, Katja Wingenfeld

**Affiliations:** 1 Department of Psychiatry and Neurosciences, Charité—Universitätsmedizin Berlin, corporate member of Freie Universität Berlin and Humboldt-Universität zu Berlin, Berlin, Germany; 2 BIH Biomedical Innovation Academy, Berlin Institute of Health (BIH) at Charité—Universitätsmedizin Berlin, Berlin, Germany; 3 Department of Cognitive Psychology, Institute of Cognitive Neuroscience, Faculty of Psychology, Ruhr University Bochum, Bochum, Germany; 4 DZPG (German Center for Mental Health), partner site Berlin, Berlin, Germany

**Keywords:** Psychiatric disorders, Pathogenesis

## Abstract

Altered fear conditioning and extinction learning are discussed as key etiological features in anxiety disorders. Women have an increased risk for anxiety disorders and fear conditioning has been shown to be influenced by the menstrual cycle phase and circulating gonadal hormones. The objective of our study was to investigate the effects of separate and combined estradiol and progesterone administration on fear extinction in healthy women. We conducted a placebo-controlled, randomized study in healthy women, who completed a fear conditioning paradigm on three consecutive days: fear acquisition training on day 1, fear extinction training on day 2, and return of fear test on day 3. Skin conductance responses (SCRs) served as main outcome variable. Two hours before testing on day 2, participants received pills containing either placebo, estradiol (2 mg), progesterone (400 mg) or the combination of both. We examined 116 women (mean age 25.7 ± 6.0 years), who showed significantly stronger conditioned SCRs to the CS+ than CS- during fear acquisition training indicating successful fear learning. At the beginning of the fear extinction training, estradiol administration reduced the differentiation between the conditioned stimuli. In the return of fear test, the estradiol groups showed heightened SCR responses to the previously extinguished stimulus, i.e., impaired extinction recall. Administration of progesterone did not have any significant influence on SCRs. There were also no effects on fear potentiated startle response. In our interpretation, exogenous estradiol administration affected the extinction of the conditioned fear response which led subsequently to a stronger return of fear. From a clinical perspective our findings suggest that estradiol levels may have an influence on the success of exposure therapy and could be taken into consideration when planning exposure sessions.

## Introduction

Fear conditioning paradigms are well-established and frequently used to study the mechanisms related to anxiety disorders [1]. There are at least two mechanisms for how fear conditioning processes mimic the development and treatment of anxiety disorders, namely enhanced acquisition of the fear response and reduced fear extinction [2]. Fear conditioning experiments in humans distinguish three distinct phases: (1) fear acquisition training, when a per se neutral cue is paired with a threat signal (unconditioned stimulus, US) and becomes a conditioned stimulus (CS) over time; (2) fear extinction training, when the CS is no longer associated with the US and finally, (3) return of fear test, when the magnitude of the reaction to the CS serves as a measure for the reoccurring fear response. After successful extinction learning, the fear reaction to the CS during the return of fear test should be significantly reduced.

Female sex is associated with a higher risk for developing stress-related disorders like anxiety disorders and post-traumatic stress disorder [3, 4]. Multiple lines of evidence from preclinical and clinical research suggest an influence of the female sex hormones estradiol and progesterone not only on learning and memory in general, but in fear conditioning in particular [5]. In turn, this influence might be one factor which could explain the sex differences in the prevalence rates observed in stress-associated disorders [3]. Preclinical fear conditioning research implies decreased fear reactions in states of high estradiol ([6, 7]) as well as high progesterone [8].

Concerning the evidence in humans, results in healthy women suggest a more complex role of hormonal influences in fear acquisition as well as extinction learning and recall [9]. Previous studies found high levels of endogenous estradiol to be associated with enhanced extinction learning (for example [5, 10, 11]). Along these lines, Merz et al. [12] demonstrated, that the intake of hormonal contraceptives was associated with decreased extinction learning compared to naturally-cycling women in the luteal phase. This result was explained by reduced levels of endogenous hormones after the intake of hormonal contraceptives. No effect of endogenous progesterone on extinction learning in humans was found so far [11, 13, 14].

Observational and quasi-experimental studies cannot disentangle the isolated effects of estradiol and progesterone. Separate administration of estradiol and progesterone reflects a more mechanistic approach to study the differential effects of both hormones on extinction training without overlap. One study examined the effect of exogenously administered estradiol (1.8 mg estrogen acetate) 30 min before extinction training in healthy, naturally-cycling women [15]. The authors found that pre-extinction estradiol administration significantly improved extinction recall on the following day. This is in line with the above-mentioned observations in states of high endogenous estradiol levels. Only a few studies regarding progesterone in humans exist and they report no significant influence of endogenous progesterone in fear conditioning [13, 14]. Felmingham et al. [16] however found in a study group of 56 healthy pre-menopausal women without hormonal contraception enhanced memory, i.e., greater memory recall under stress for negative images during high progesterone levels. Up to now, there is no published study using exogenous progesterone in fear conditioning. However, assuming an involvement of progesterone in human fear conditioning might be reasonable. In an fMRI study, van Wingen and colleagues [17] could show that a onetime administration of oral progesterone in healthy young women in the follicular cycle phase was associated with an increased amygdala reactivity (however without an effect on state anxiety and mood). Furthermore, van Wingen and colleagues [18] also showed that progesterone decreased responses to faces in the amygdala during memory encoding.

To sum up, there is some evidence for beneficial effects of endogenous estradiol in extinction learning and there is a rather large body of convincing evidence supporting a beneficial effect of endogenous estradiol on extinction recall [10, 11, 13–15, 19–21]. Moreover, preclinical research supports the notion that high endogenous progesterone levels might enhance the observed beneficial effects [8]. Graham et al. [22] conducted research in ovariectomized female rats, who were either treated with exogenous administered β-estradiol or the combination of β-estradiol and progesterone. They found that β-estradiol alone improved extinction recall and that the addition of progesterone (with extinction training happening within 6 h after administration) even augmented this positive effect. Along these lines, naturally cycling female rats showed improved extinction consolidation after having been injected the combination of estradiol and progesterone before extinction learning during the metestrus phase with naturally low estrogen and progesterone levels [23]. However, so far there is no study examining the role of exogenously administered progesterone in humans in fear conditioning. Furthermore, the determination of the cycle phase in earlier studies was mainly based on self-report, which might be misleading. Therefore, in this study we systematically examined the differential effects of single and combined estradiol and progesterone administration on fear extinction in healthy, naturally-cycling women. Moreover, besides self-report we also measured hormone levels in saliva. Based on previous research [15] we hypothesized that applying estradiol before extinction training would improve extinction learning and consequently lead to a reduced fear response during the return of fear test. Based on preclinical research in female rats, we assumed that this effect would be augmented by adding progesterone. In other words, fear reactions following stimuli presentation should not differ between groups during fear acquisition training and extinction training, but during the return of fear test: pre-extinction estradiol administration compared to placebo should be associated with less pronounced fear reactions during the return of fear test.

## Methods and materials

### Participants

We recruited a total of 116 healthy pre-menopausal women (no psychiatric disorders, gynecological and endocrinological diseases). All participants had to have a regular menstrual cycle. The definition of a regular menstrual cycle was based on the National Institute of Child Health and Human Development’s statement “About Menstruation” as well as Dasharathy et al. [24], with an average menstrual cycle lasting for 28 days (time span: 21–35 days) and including an average menstruation time of about 5 days. All women were tested in the follicular cycle phase, which was based on self-report regarding their last onset of the menstrual bleeding. No intake of hormones (including hormonal contraception) and medication interfering with the hypothalamic-pituitary-gonadal axis were allowed. Moreover, women during gestation and lactation were not included in the study. Before inclusion, all participants had to provide written informed consent.

### Procedure

**Fig. 1:**
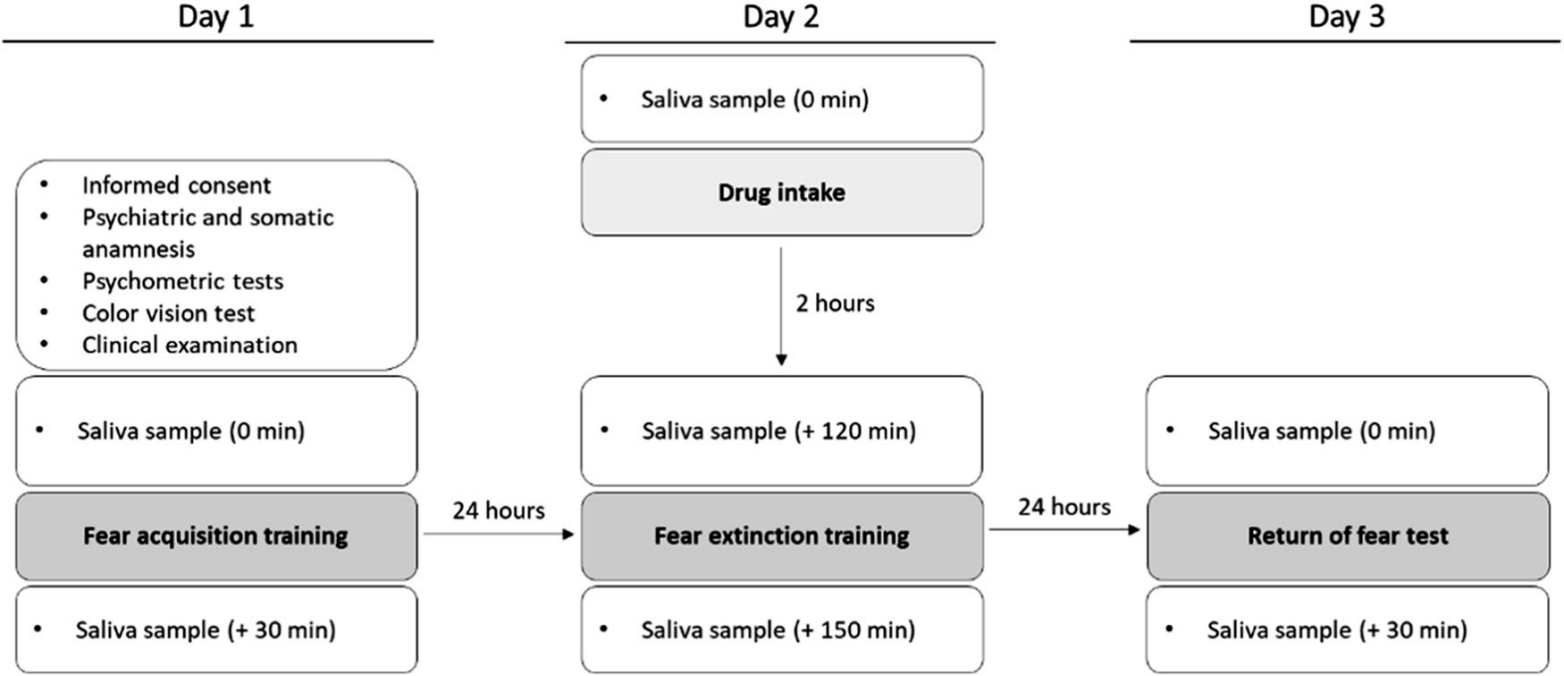
Schedule of the testing days.

The study took place at the Department of Psychiatry and Neurosciences, Campus Benjamin Franklin of the Charité—Universitätsmedizin Berlin. Participants were recruited from December 2019 to January 2022 by using public and online postings. At the beginning, all participants underwent an extensive screening procedure including the record of any psychiatric, gynecological as well as endocrine diagnoses. After pre-screening for inclusion and exclusion criteria, participants were invited into the clinic and a general medical history was taken. All participants underwent a clinical interview in order to check for current and lifetime psychiatric disorders (M.I.N.I.; [25]) as well as a clinical (including gynecological and endocrinological) history. After completion, all participants filled out self-rating measures for depressive symptoms (Beck Depression Inventory; [26]), trait anxiety (STAI-T; [27]), and a color vision test [28] to ensure that the participants could correctly recognize the colors used in the fear conditioning paradigm. Finally, all participants underwent a clinical examination, an electrocardiogram, and a urine pregnancy test. We implemented a randomized, single-blind, placebo-controlled, between-subjects design. The Declaration of Helsinki was adhered to. The ethics committee of the Charité approved the study. All participants received a onetime monetary remuneration of up to €120. Figure 1 displays the three testing days.

#### Fear conditioning paradigm

We used a fear conditioning paradigm adopted from Milad et al. [29]. Participants were seated in front of a standard 22-inch monitor and prepared for testing. Two solid-gel electrodes were positioned on the back of the right hand for the application of an electric stimulation serving as the unconditioned stimulus (US), additionally, the electrodes for psychophysiological recordings were positioned. To determine the subjective threshold for the US, which was supposed to be aversive but not painful, gradual adjustment of the electric voltage (10–120 V) was performed before the testing started. The experiment was subsequently performed using each participant’s individual threshold value on day 1 and again on day 3.

**Fig. 2:**
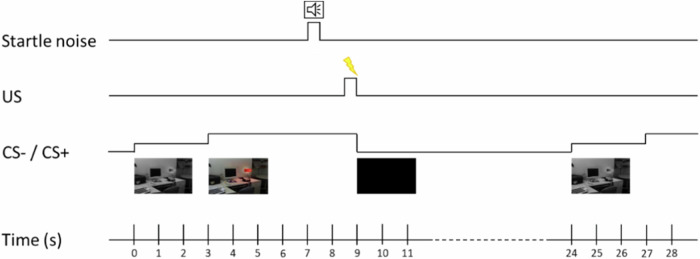
Timeline of the conditioning paradigm. Participants saw photographs containing a lamp, which switched on after 3 s and glowed in either yellow, blue, or red for another 6 s (CS). Startle noise was presented 6.5–7 s after CS onset, followed by an electric stimulation (US) in 62.5% of the cases, CS conditioned stimulus.

Participants saw photographs (office room on day 1, office shelf on days 2 and 3) containing a lamp, which was switched off at the beginning. After 3 s the lamp switched on and glowed in yellow, blue, or red for another 6 s, which served as the conditioned stimulus (CS). This was followed by an inter-trial interval (black screen) of 15 s (Fig. 2). Throughout the whole study, each color was counterbalanced across CS-, the extinguished (CS + E), and the unextinguished stimulus (CS + U).

During fear acquisition training on day 1, each color was presented 8 times in total (starting right after a preconditioning phase without the US). Two of the three colors were followed by the US in 62.5% of the presentations (and thus became the CS + E and the CS + U). The first and the last presentation of the CS + E and CS + U was always followed by the US. The third light was never paired with the US (CS-). No more than two of the same stimuli were presented in a row. After fear acquisition training, all participants were asked to name the colors, which were associated with the US in order to ensure contingency awareness. On day 2, CS- and CS + E (but not the CS + U) were presented 5 times but without any US for fear extinction training. For the return of fear test on day 3, again all three CS were presented 5 times each without an electric stimulation. For fear reinstatement, each participant afterwards received four electric stimuli (using the individual electric voltage from day 1) in front of a gray monitor screen, which was shown for 20 s. During the following fear reinstatement test, presentation of the CS was repeated in the same manner as for the return of fear test.

Two solid-gel electrodes (Tyco H34SG), positioned in the palm of the left hand, were used for the recording of SCRs, two Tyco Arbo H124SG electrodes were placed right below the left lower eyelid to record the startle response. Startle stimuli were acoustic white noise probes (105 dB, 50 ms duration, instantaneous rise time, binaural stimulation) presented via audiometric headphones (Holmco PD-81, Holmberg GmbH & Co. KG, Germany). Psychophysiological data were analyzed following the protocol as in Kuehl et al. [30]. Natural log-transformed SCRs and startle responses served as outcome variables on all 3 days. SCRs were measured using the BIOPAC MP 150 and the GSR100 Amplifier and analyzed using Ledalab (version 3.4.9; [31]). Only responses with an amplitude ≥0.01 µS during the first 1 to 4 s after CS presentation were scored [32]. Startle noise onset happened 6.5 to 7 s after CS onset. On days 2 and 3, the startle noise was additionally presented six times before the paradigm started. The startle responses (difference between peak (20 to 150 ms after noise onset) and baseline signal (50 ms prior to noise onset)) were recorded on hard disk using the BIOPAC MP 150 and the EMG 100 C amplifier at 16 bit resolution and with a 1 kHz sampling rate. Hardware band-pass filter settings were 10 to 500 Hz, followed by a 28 Hz software high-pass filter [33]. The raw signal was rectified and integrated online with a time constant of 10 ms [34] and analyzed offline with a C++based, semi-automated program. Each response was manually confirmed. Non-responses were set to zero and included in the analysis, whereas artifacts (for example spontaneous eye blinks coinciding with the stimulus) were excluded from the analysis.

#### Pharmacological intervention

Two hours before the start of extinction training on day 2, all participants received three pills in a blinded manner. By combining placebo, estradiol, and progesterone, each participant was randomized to one of the four treatment conditions: (1) placebo + placebo, (2) placebo + estradiol, (3) placebo + progesterone, (4) estradiol + progesterone. Both estradiol groups received 2 mg estradiolvalerat (Gynokadin®), a dosing based on Graham and Milad [15] as well as Bayer et al. [35], who could show that 2 mg were associated with a marked increase in salivary estradiol concentrations as well as enhanced hippocampal activity. The progesterone groups received in total 400 mg of micronised progesterone (Utrogest®; 200 mg per pill) as in van Wingen et al. [18], who found an increase in hippocampus responses [17, 18, 36]. Estradiol peaks 2 to 4 h and progesterone 1 to 3 h after administration (based on serum levels; respective summaries of product characteristics for the used drugs by the pharmaceutical company DR. KADE Pharmazeutische Fabrik GmbH). Pre-menopausal women without intake of hormonal contraceptives show estradiol in saliva samples to be in a range between 3.1 and 6.4 pg/ml (=11.4–23.5 pmol/l) during the follicular cycle phase. During midcycle, women show increased mean saliva estradiol concentrations of 4.9–11.9 pg/ml (=17.98–43.67 pmol/l; see instructions for use of IBL International’s enzyme immunoassay for the quantitative determination of 17beta-Estradiol in human saliva). As for progesterone, the reported range was between 30.3 and 51.3 pg/ml (=111.2–188.3 pmol/l) for pre-menopausal women in the follicular phase. During the luteal phase, progesterone increased to 87.3–544.3 pg/ml (=320.39–1997.58 pmol/l; see instructions for use of IBL International’s Progesterone Saliva ELISA).

#### Measuring of saliva hormone concentrations

On day 1 and 3, saliva samples to measure saliva estradiol and progesterone levels were assessed before (0 min) and after testing (+30 min) using salicaps. On day 2, saliva samples were taken before medication intake (0 min) as well as before (+120 min) and after testing (+150 min).

For all endocrine analyses, we used enzyme-linked immunosorbent assays (ELISA; IBL International GmbH, Germany). All samples and standards were measured in duplicate, and the detection limit for 17beta-estradiol was 2.1 pg/ml, and for progesterone 3.13 pg/ml. The intra- and inter-assay coefficients of variation for estradiol were lower than 8.8 and 11.8%, respectively. The intra- and inter-assay coefficients of variation for progesterone were lower than 4.9 and 6.7%, respectively.

### Statistical analyses

For the analyses of demographic, clinical, and psychometric data, we used *univariate ANOVAs* for continuous variables and *Chi²-tests* for categorical variables.

To examine changes in estradiol and progesterone concentrations we ran separate *repeated measures ANOVAs* with two (for day 1 and 3) or three time points (for day 2; before hormone intake, before testing, after testing) as within-subjects factor and treatment (respective hormone yes or no) as between-subjects factors.

Since the CS were presented in a mixed order, all trials were aggregated into separate blocks:

All 24 trials of day 1 were aggregated into four blocks containing two presentations of each CS in order to establish a balanced distribution of all CS within one block (i.e., six trials per block). Day 2 had a total of 10 trials (five trials of the CS- and five trials of the CS + E). In order to ensure an equal distribution between CS + E and CS- per block we aggregated the first six trials into block 1 (three CS- presentations and three CS + E presentations) and the last four trials into block 2 (two CS- presentations and two CS + E presentations). For day 3, we aggregated all 15 trials into two blocks for the return of fear test (block 1: always the first three presentations of CS-, CS + E, and CS + U; block 2: always the remaining two presentations of CS-, CS + E and CS + U). The same sequence was repeated immediately afterward for the fear reinstatement test (therefore day 3 consisted of four blocks in total).

For the analysis of day 1, we used a *repeated measures* 4 × 3 *ANOVA* with the four blocks and the three CS as within-subjects factors. Additionally, we reran this analysis with estradiol (yes/no) and progesterone (yes/no) as between-subjects factors to check for baseline differences between the four treatment groups. For the analysis of day 2, we used a *repeated measures* 2 × 2 × 2 × 2 *ANOVA* with two blocks and two CS as within-subjects factors as well as estradiol (yes/no) and progesterone (yes/no) as between-subjects factors. Finally, for the analysis of day 3, we used a *repeated measures* 4 × 3 x 2 × 2 *ANOVA* with four blocks and the three CS as within-subjects factors as well as estradiol (yes/no) and progesterone (yes/no) as between-subjects factors.

A *p* ≤ 0.050 was considered statistically significant. Degrees of freedom were corrected using Greenhouse-Geisser estimates of sphericity. Bonferroni-corrected *post-hoc t-tests* were applied to determine SCR differences. Partial *η*^*2*^ was used as the measure of effect size. All analyses were performed using IBM SPSS Statistics (version 28).

## Results

### Sociodemographic, clinical, and psychometric data

All sociodemographic, clinical, and psychometric data of the sample are presented in Table 1. Overall, the sample consisted of 116 young, normal weight, well-educated women (please see Fig. S1 in the Supplemental Materials for a CONSORT diagram). The mean length of the menstruation cycle was 29.5 (±2.8) days above all groups. In concordance with the inclusion criteria, no participant had a current or lifetime mental disorder. Accordingly, psychometric scores, i.e., BDI and STAI, yielded no clinically relevant results. All participants were randomized to one of the four treatment groups: (1) placebo, (2) estradiol, (3) progesterone, (4) estradiol and progesterone. There were no statistically significant differences between the four treatment groups concerning any of the variables.

**Table 1:** Sociodemographic, clinical, and psychometric data.

		Placebo	Estradiol	Progesterone	Estradiol + Progesterone	
		*N* = 30	*N* = 29	*N* = 28	*N* = 29	*statistics*
Sociodemographic and clinical data						
Age (years)	*M* (*SD*)	26.0 (6.1)	25.2 (6.3)	25.8 (6.3)	25.7 (5.8)	*F*(3, 112) = 0.096, *p* = 0.962
Education (years)	*M* (*SD*)	12.2 (0.9)	12.2 (1.1)	12.2 (0.8)	12.4 (0.5)	*F*(3, 112) = 0.354, *p* = 0.786
Smoker (yes)	*N* (%)	6 (20.0)	6 (20.7)	5 (17.9)	6 (20.7)	*Χ*^*2*^(3) = 0.096, *p* = 0.992
BMI (kg/m^2^)	*M* (*SD*)	21.9 (2.3)	22.0 (2.7)	22.3 (3.0)	22.6 (2.6)	*F*(3, 112) = 0.335, *p* = 0.800
Length of menstruation cycle (days)	*M* (*SD*)	29.4 (2.5)	29.4 (3.6)	29.5 (2.9)	29.5 (2.3)	*F*(3, 112) = 0.007, *p* = 0.999
Duration between menstruation and testing (days)	*M (SD)*	2.8 (2.5)	3.5 (2.2)	3.7 (2.2)	2.7 (2.1)	*F*(3, 112) = 1.311, *p* = 0.275
Psychometric data						
BDI (sum score)	*M* (*SD*)	2.3 (2.6)	2.0 (2.2)	2.1 (2.5)	2.3 (2.9)	*F*(3, 112) = 0.106, *p* = 0.956
STAI-T (sum score)	*M* (*SD*)	42.1 (2.7)	42.2 (5.2)	42.5 (3.8)	42.0 (4.9)	*F*(3, 112) = 0.070, *p* = 0.976

*BMI* body mass index, *BDI* Beck Depression Inventory, *STAI-T* State-Trait Anxiety Inventory-Trait anxiety. Depiction of means (*M*) and standard deviations (*SD*) as well as number (*N*) in %.

### Saliva concentrations of estradiol and progesterone

We found no significant differences in estradiol and progesterone concentrations among the four treatment groups at day 1 and 3, neither before nor after testing (all *p* > 0.050).

**Fig. 3:**
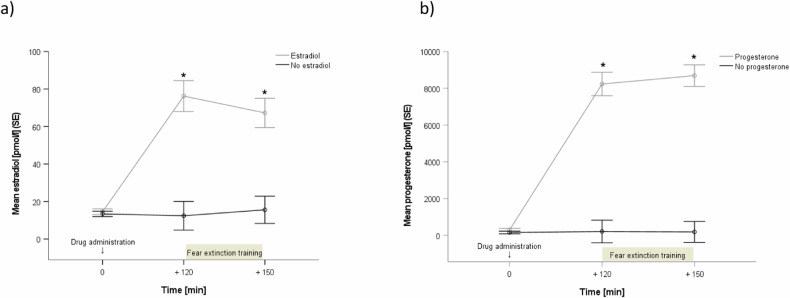
Manipulation check. Depiction of saliva estradiol (**a**) and progesterone (**b**) concentrations on day 2 before drug intake (0 min) as well as before (+120 min) and after fear extinction training (+150 min). A significant time by treatment interaction indicated significantly higher estradiol and progesterone concentrations after the respective drug administration (2 mg estradiol; 400 mg progesterone). (For a depiction of saliva estradiol and progesterone concentrations on day 1, day 2, and day 3 broken up for all treatment groups, see Fig. S2 in the Supplemental Materials).

As manipulation check (day 2), we compared estradiol and progesterone levels before and after drug administration by using a *3* × *2 repeated measures ANOVA* with time (three-time points) and treatment (respective hormone yes or no). For estradiol, we found a significant main effect of time (*F*(1.68, 159.56) = 21.1, *p* < 0.001, *pη*^*2*^ = 0.18), treatment (*F*(1, 95) = 32.9, *p* < 0.001, *pη*^*2*^ = 0.25), and time by treatment interaction (*F*(1.68, 159.56) = 20.8, *p* < 0.001, *pη*^*2*^ = 0.18). *Post-hoc t-tests* revealed a significant increase of the saliva estradiol concentrations 120 and 150 min after drug intake compared to pills without estradiol (both *p* < 0.001) (Fig. 3a). Mean estradiol level at baseline was 12.7 pmol/l, increasing to 82.6 pmol/l 150 min after hormone intake.

For progesterone, the same pattern was observed: a significant main effect of time (*F*(1.85, 207.25) = 69.7, *p* < 0.001, *pη*^*2*^ = 0.38), treatment (*F*(1, 112) = 112.7, *p* < 0.001, *pη*^*2*^ = 0.50), and time by treatment interaction (*F*(1.85, 207.25) = 68.3, *p* < 0.001, *pη*^*2*^ = 0.37) emerged. *Post-hoc t-tests* again revealed a significant increase of the saliva progesterone concentration 120 and 150 min after drug intake compared to pills without progesterone (both *p* < 0.001) (Fig. 3b). Mean progesterone level at baseline was 208.8 pmol/l, increasing to 8099.1 pmol/l 150 min after hormone intake. In sum, our study population showed hormone values in the range reported for the follicular cycle phase on day 1, and showed an increase, which was above the physiological range, e.g., at midcycle of the luteal phase on day 2.

### Fear conditioning

#### Skin conductance responses

##### Fear acquisition training (Day 1)

**Fig. 4:**
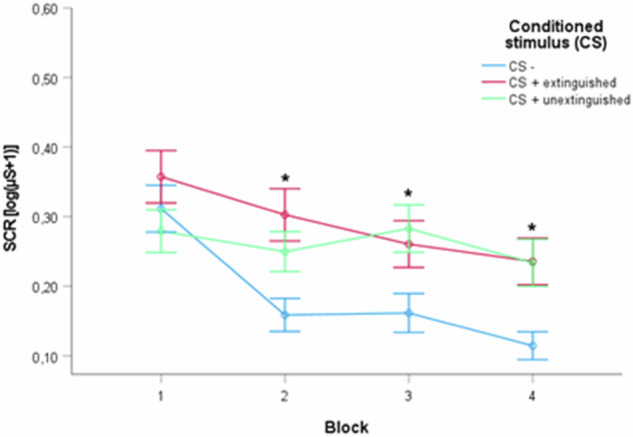
Fear acquisition training across the whole sample. Depiction of the SCRs regarding the three conditioned stimuli (CS-,CS+E, CS+U) for the whole sample over the course of four blocks. Both CS+ were associated with significantly higher SCRs compared to the CS- in blocks 2, 3, and 4. There were no significant differences in SCRs between the two CS+. Significant differences are marked (*p < 0.001). (For a depiction of the SCRs regarding the three conditioned stimuli over the course of four blocks during fear acquisition training on day 1 broken up into the four treatment groups see Fig. S3 in the Supplemental Materials).

An *ANOVA* with time and stimulus revealed a significant main effect of time (*F*(2.3, 265.8) = 15.06, *p* < 0.001, *pη²* = 0.12), a significant main effect of stimulus (*F*(1.8, 201.2) = 19.128, *p* < 0.001, *pη²* = 0.14) and a significant time by stimulus interaction (*F*(5.4, 616.5) = 3.711, *p* = 0.002, *pη²* = 0.03). *Post-hoc t-tests* revealed significant SCR differences between both CS+ (the stimuli with the electric stimulation) and the CS- (the stimulus without the electric stimulation) in block 2, block 3, and block 4 (all *p* < 0.001 in all CS + E vs. CS- and CS + U vs. CS- comparisons), but not block 1 (CS + E vs. CS-: *t* = −1.356, *p* = 0.178; CS + U vs. CS-: *t* = 1.118, *p* = 0.266) indicating significantly stronger SCR responses after both CS+ compared to the CS- over time. There was no significant SCR difference between both CS+ (Fig. 4). Breaking up the whole sample into the four treatment groups revealed no significant SCR differences at baseline and in response to the respective CS (all *p* > 0.050).

Moreover, 94.0% of all participants could correctly name the CS- from both CS+ after completion of day 1 (there were no significant differences between the four treatment groups), indicating a high rate of contingency awareness. Taken together, fear acquisition was successful.

##### Fear extinction training (Day 2)

**Fig. 5:**
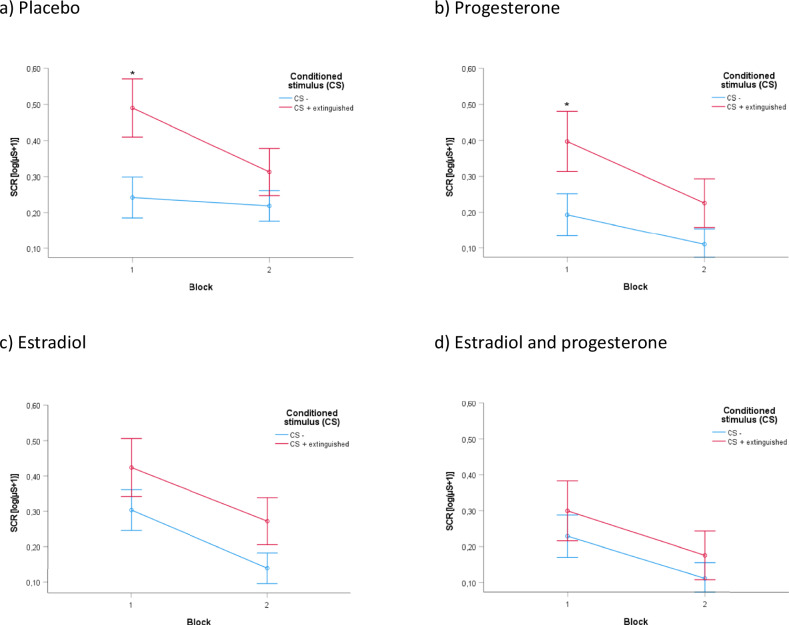
Fear extinction training in the four treatment groups. Depiction of the SCRs towards the CS + E and the CS- for the four distinct treatment groups. Significant SCR differences between the CS + E and the CS- were found only in conditions without estradiol (*p < 0.001), but not after estradiol administration in block 1. No significant SCR differences appeared in block 2.

An *ANOVA* with the within-subject factors time and stimulus and the between-subjects factors estradiol (yes, no) and progesterone (yes, no) revealed a significant main effect of time (*F*(1.0, 111.0) = 39.776, *p* < 0.001, *pη²* = 0.26), stimulus (*F*(1.0, 111.0) = 16.173, *p* < 0.001, *pη²* = 0.13), time by stimulus interaction (*F*(1.0, 111.0) = 4.059, *p* = 0.046, *pη²* = 0.04) as well as a significant time by stimulus by estradiol interaction (*F*(1.0, 111.0) = 4.489, *p* = 0.036, *pη²* = 0.04). There was no significant effect of progesterone and no significant estradiol by progesterone interaction. In block 1, *post-hoc t-tests* across groups revealed significant SCR differences between the CS + E and the CS- only in the groups without estradiol (*t* = −4.282, *p* < 0.001) (Fig. 5a, b), but not after estradiol administration (*t* = −1.746, *p* = 0.043; not significant after Bonferroni correction) (Fig. 5c, d) (see also Table S1 in the Supplementary). In block 2, there were no significant SCR differences. No other explorative post-hoc comparisons revealed significance. Taken together, higher SCRs after CS + E compared to CS- occurred only in the groups receiving no estradiol, whereas estradiol administration reduced the CS + E/CS- differentiation. Figure 5 displays all treatment conditions separately.

##### Return of fear and fear reinstatement test (Day 3)

An *ANOVA* with the within-subjects factors time and stimulus and the between-subjects factors estradiol (yes, no) and progesterone (yes, no) revealed a significant main effect of time (*F*(2.3, 265.2) = 47.613, *p* < 0.001, *pη²* = 0.30), stimulus (*F*(1.9, 218.1) = 11.504, *p* < 0.001, *pη²* = 0.10) and time by stimulus interaction (*F*(4.9, 535.7) = 6.612, *p* < 0.001, *pη²* = 0.06).

**Fig. 6:**
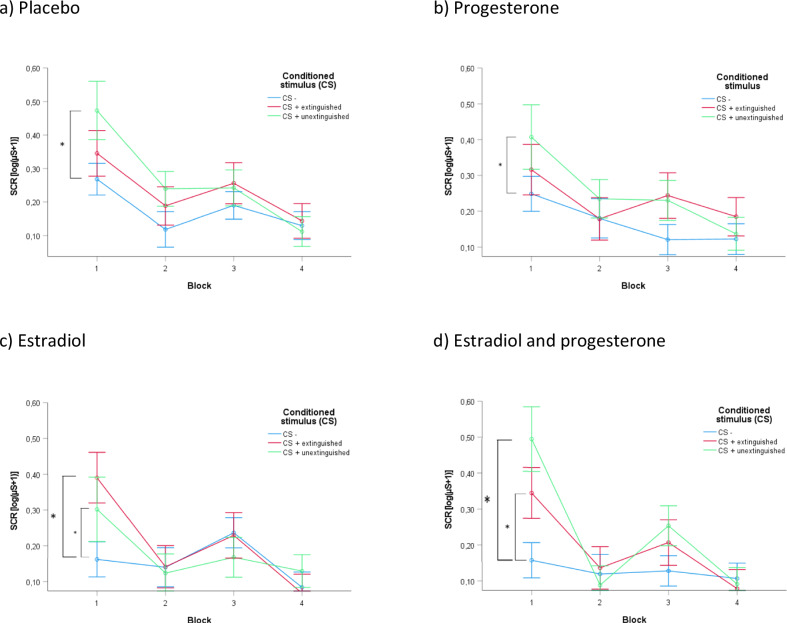
Return of fear (blocks 1 and 2) and fear reinstatement test (blocks 3 and 4) in the four treatment groups. Depiction of the SCRs towards the CS + E, CS + U, and CS- in the fourdistinct treatment groups. Blocks 1 and 2 represent return of fear test, blocks 3 and 4 show the SCRs after fear reinstatement. We found a significant ‘time by stimulus by estradiol effect’, which again was broken down for ‘block’ and ‘estradiol’. SCR responses to the CS + U were significantly stronger compared to the CS- in block 1, independent of estradiol. Only after estradiol, we additionally found SCRs following CS + E to be significantly higher than those to the CS-. There were no significant SCR differences in blocks 2 to 4. (*p < 0.001, of note, we did not calculate individual comparisons separately for “progesterone” since this factor did not significantly contribute to any interaction, the two by two depiction for both treatment conditions serves a purely illustrative purpose).

Importantly, we found a significant time by stimulus by estradiol interaction (*F*(4.9, 535.7) = 2.516, *p* = 0.03, *pη²* = 0.02). To further investigate this effect, we broke down the analysis block-wise and calculated contrasts between CS- vs. CS + E and CS- vs. CS + U, separate for each block and for estradiol vs. no estradiol. For the comparison of estradiol vs. no estradiol, we found a significant difference between the CS + E and the CS- (*t* = −4.760, *p* < 0.001), indicating impaired extinction recall (i.e., heightened SCR responses) after having received estradiol before fear extinction training. Participants that had not received estradiol did not show a significant difference between the CS + E and the CS- (*t* = −1.91, *p* =0.061), suggesting effective extinction recall. Across treatments, responses to CS + U were significantly stronger compared to CS- in block 1 (without estradiol (Fig. 6a, b): *t* = −3.315, *p* < 0.001; with estradiol (Fig. 6c, d): *t* = −4.905, *p* < 0.001). There were no significant differences between the CS and the treatment groups in blocks 2 to 4, i.e., during late return of fear and the fear reinstatement test. Taken together, these findings indicate a specific estradiol effect on the CS + E without transfer to the CS + U. There was no significant effect of progesterone and no significant estradiol by progesterone interaction. Figure 6 displays all treatment conditions separately, more detailed post-hoc analyses are presented in the supplement (Table S2).

#### Fear-potentiated startle

Analyzing the left lower eyelid EMG instead of the SCRs as outcome variable revealed a significant main effect of the stimulus during fear acquisition training (*F*(2.0, 227.1) = 23.385, *p* < 0.001, *pη²* = 0.17). *Post-hoc t-tests* for stimulus revealed significant differences between both CS+ and the CS- (CS + E vs. CS-: *t* = −5.464, *p* < 0.001; CS + U vs. CS-: *t* = −6.131, *p* < 0.001), but not between both CS+ (*t* = −0.938, *p* = 0.175).

Regarding extinction training on day 2, we again found a significant main effect of stimulus (*F*(1, 106) = 30.777, *p* < 0.001, *pη²* = 0.23), but no main or interaction effect of estradiol or progesterone. *Post-hoc t-tests* for stimulus only revealed a significant difference between the CS + E and the CS- (*t* = −5.431, *p* < 0.001).

Finally, concerning the return of fear and fear reinstatement test on day 3, we found a significant main effect of stimulus (*F*(2.0, 213.3) = 12.419, *p* < 0.001, *pη²* = 0.10), but again no main or interaction effect of estradiol or progesterone. *Post-hoc t-tests* for stimulus revealed a significant difference between both CS+ and the CS- (CS + E vs. CS-: *t* = −3.856, *p* < 0.001; CS + U vs. CS-: *t* = −5.064, *p* < 0.001).

## Discussion

We investigated the effects of separate and combined estradiol and progesterone administration on extinction learning and recall in healthy pre-menopausal women. We assumed that applying estradiol before fear extinction training would improve extinction learning and consequently lead to a reduced SCR response (i.e., stronger reduction in conditioned responses compared to placebo) during the return of fear test, which would be further augmented by adding progesterone. After estradiol administration however, we unexpectedly observed no SCR differences between CS during fear extinction training, which was clearly seen in the placebo and progesterone only groups. This finding is striking, because it indicates that estradiol administration was first of all associated with a reduced SCR reaction regarding the to be extinguished CS. This stands in contrast to the pattern on the following day: SCRs to the extinguished CS were significantly higher compared to the CS- in the estradiol groups. However, participants that were not administered estradiol exhibited SCRs to the extinguished CS in the return of fear test that did not significantly differ from responses to CS-, indicative of a successful extinction. Progesterone did not have any effects on extinction learning and recall. The responses to the unextinguished CS+ (CS + U) in the return of fear test were not influenced by the experimental manipulation, i.e., the post-hoc comparison CS- vs. CS + U was significant for all manipulation combinations. Thus, the previously described effect of estradiol on the CS + E did not generalize to the CS + U.

The manipulation check revealed significant increases in estradiol and progesterone concentrations during extinction training, whereas, before the return of fear test, sex hormone concentrations went back to the basic levels observed during fear acquisition training, indicating a successful treatment. Baseline saliva levels of estradiol and progesterone were within the range reported for women in the follicular cycle phase. After drug administration the increase in saliva estradiol and progesterone was above the physiological range of all menstrual cycle phases. However, these values were comparable with other studies (e.g., [18, 35]). Therefore, we worked with supraphysiological hormone levels during extinction training.

Against the background of the scarcity of studies using exogenous estradiol, our finding is interesting in two ways. First, by showing that estradiol administration is associated with effects on extinction learning and recall, we add to the evidence of estradiol plays a significant role in fear conditioning in humans. Secondly, we found two main results concerning estradiol, which happens to be the opposite of what we expected: (1) after estradiol administration—and in contrast to participants that did not receive estradiol—participants insufficiently discriminated between the CS, i.e., the responses between the CS + E and CE- did not significantly deviate from the beginning on, an effect which we would interpret as impaired retrieval of the information learned on the previous day since the conditioning procedure clearly was successful in these participants as well and (2) on day 3, participants having received estradiol before fear extinction training showed a significantly stronger return of fear towards the extinguished CS indicating a lack of fear reduction. We assume that the latter is a consequence of the previous result: During extinction learning the no-estradiol groups showed significant SCR differences only in block 1, but not in block 2, which is indicative of successful extinction learning. This was, however, not found in the estradiol groups, where the participants failed to show significant SCR differences at the very beginning of the experiment. This finding could represent a problem in differentiating between stimuli rather than learning per se. Taking into account, that all groups (meaning the estradiol groups as well) significantly differentiated all CS at the end of fear acquisition training on day 1, our results indicate a diminished recall of the information regarding the CS + E learned on the previous day. In other words, the lack of differentiation between both CS already at the beginning of the extinction training suggests the interpretation, that especially the recall of the conditioned information from day 1 was impaired by estradiol. According to this interpretation, the influence of estradiol would be less on (un)learning during extinction, but rather on retrieval. Since the information of the to be extinguished CS was impaired in its retrieval, it was to a certain extent protected from this extinction (thus reappearing during the return of fear test).

When looking at the study by Graham and Milad [15], where estradiol had no effect on extinction learning, but improved extinction recall, the only conceptual difference is the time point of estradiol administration. Graham and Milad also performed a manipulation check by assessing serum estradiol levels right before drug intake and after extinction training on day 2 and found significantly elevated estradiol levels right after completion of the extinction training (as we have). However, our participants received the blinded pills 120 min before fear extinction training, whereas Graham and Milad administered estradiol only 30 min before extinction training. This might be interpreted as a time-dependent effect of estradiol. Correspondingly, estradiol not only exerts its effects via slow genomic pathways taking hours [37] but also by binding to membrane-bound receptors, resulting in fast non-genomic effects occurring within minutes [38, 39]. Another explanation focuses on the different phases of learning: We found, that estradiol and progesterone reached peak salivary levels 120 min after administration of the pills. This was when we ran the fear extinction training. Graham and Milad administered the hormones 30 min before extinction training. Estradiol might not have influenced learning in their experiment, but rather consolidation of the information, whereas we assume that in our experiment estradiol influenced retrieval of information from the previous day. Another possible explanation derives from an emerging body of preclinical research describing the time-dependent effects of estradiol as well as progesterone on dopaminergic signaling in the dorsal striatum and nucleus accumbens in female rats [40]. Basically, estradiol is supposed to increase dopamine release right after its administration, while progesterone can even potentiate this effect. In the further course, however, an inhibition of dopamine release is detected. This was observed in essentially the same manner in ovariectomized rats as well as in naturally-cycling female rats [22]. Future studies should therefore address the question of time-dependent effects of sex hormones in humans by running fear extinction trainings for example 30, 60, 120, and more minutes after hormone administration and also targeting dopamine levels as potential readout. In particular, information regarding the optimal time point of estradiol administration becomes relevant for studies examining the potential of estradiol augmented exposure therapy in patients with post-traumatic stress disorder [41]. Finally, in adult female rats Graham and Scott [42] found that the administration of exogenous estradiol before extinction training showed a dose-dependent effect: lower as well as higher estradiol levels were associated with impaired extinction recall. One could assume, that due to the time difference after administration, estradiol levels in our study were higher compared to those in Graham and Milad [15].

To the best of our knowledge, this study is the first to examine the influence of exogenous progesterone on fear conditioning in humans revealing no effects of progesterone on extinction learning and recall. This null result is in line with research on endogenous progesterone also having no significant consequences on learning and memory in humans [13, 14]. However, previous work has found an influence of exogenous progesterone on amygdala activity, a brain region specifically related to fear and anxiety. Van Wingen and colleagues [17] could show that a onetime administration of oral progesterone in healthy young women in the follicular cycle phase was associated with an increased amygdala reactivity (without an effect on state anxiety and mood). Furthermore, van Wingen and colleagues [18] showed that progesterone decreased responses to faces in the amygdala during memory encoding. Finally, even though progesterone seems to exert some function alone and in combination with estradiol in rats [8], it remains unclear in how far this is also the case in humans.

Finally, comparing both outcome measures (SCRs and fear-potentiated startle) we found converging results with regard to the discrimination between CS. However, the effects of estradiol were not significantly detected using the fear-potentiated startle. This contradiction might be reconciled by taking into account the slight, but important differences of the measured physiological processes. As described by Leuchs et al. [43], SCRs are primarily interpreted as a general arousal response, whereas fear-potentiated startle is supposed to be more of a measure of the affective component of learning processes. In our experimental setup we can therefore only say that estradiol administration had an effect on arousal-related constructs such as SCRs.

Our study had several strengths and limitations. We examined a relatively young, well-educated, and per se healthy group of women, thus, a homogeneous group. However, this advantage also comprises a limitation, because our results cannot be extrapolated to older (especially post-menopausal) women, women taking hormonal contraceptives, and women with mental disorders like anxiety disorders or post-traumatic stress disorder. Although we measured salivary hormone levels, we were only able to base our time points of testing on participant reports about their last menstruation and could therefore not distinguish between late luteal and early follicular phases as well as early and late follicular phase with absolute certainty. However, testing happened on average two to three days after menstruation, and hormone levels on days 1 and 3 and baseline on day 2 were comparably low and not significantly different from each other, which supports the notion that testing happened as planned during the follicular phase. Moreover, our salivary mean levels of estradiol and progesterone before drug intake were in the expected range of healthy pre-menopausal women in the follicular cycle phase. The increase to supraphysiological levels after drug intake, however, could be interpreted as a potential limitation because they exceeded estradiol and progesterone levels during the luteal phase by far. Additionally, progesterone administration has been shown to also increase levels of allopregnanolone, which has also been associated with effects on hippocampal and amygdala activation [18]. Future studies, thus, should also measure allopregnanolone levels. Regarding fear-potentiated startle, our startle EMG analysis lacks a noise-alone baseline, which is typically a key component of establishing fear potentiation. Finally, the single-blind design would be improved by implementing a double-blind hormone administration.

To conclude, we were able to demonstrate a significant detrimental effect of estradiol on the retrieval of information during extinction learning. This adds to the growing body of literature on sex hormones and provides multiple directions for future studies. These should consider varying time-dependent effects of sex hormones and the inclusion of participants using hormonal contraceptives as well as post-menopausal women. From a clinical perspective, our findings suggest that estradiol levels may have an influence on the success of exposure therapy and could be taken into consideration when planning exposure sessions.

## Supplementary information

FINAL SUBMISSION_Fear Conditioning_Supplemental material_2024.08.21

## Data Availability

The data of this study are available from the authors upon reasonable request (please contact MK; e-mail address: michael.kaczmarczyk@charite.de). The data are not publicly available because of legal and ethical restrictions.
