# Supplementary material for: Effects of separate and combined estradiol and progesterone administration on fear extinction in healthy pre-menopausal women
Source: Transl Psychiatry. 2024 Oct 24;14:449. doi: 10.1038/s41398-024-03079-4 (PMC11502897; doi:10.1038/s41398-024-03079-4)
Supplement: Supplementary file 1 — FINAL SUBMISSION_Fear Conditioning_Supplemental material_2024.08.21 [file 41398_2024_3079_MOESM1_ESM.docx]

**Supplemental material**

**Figure S1**

CONSORT diagram.


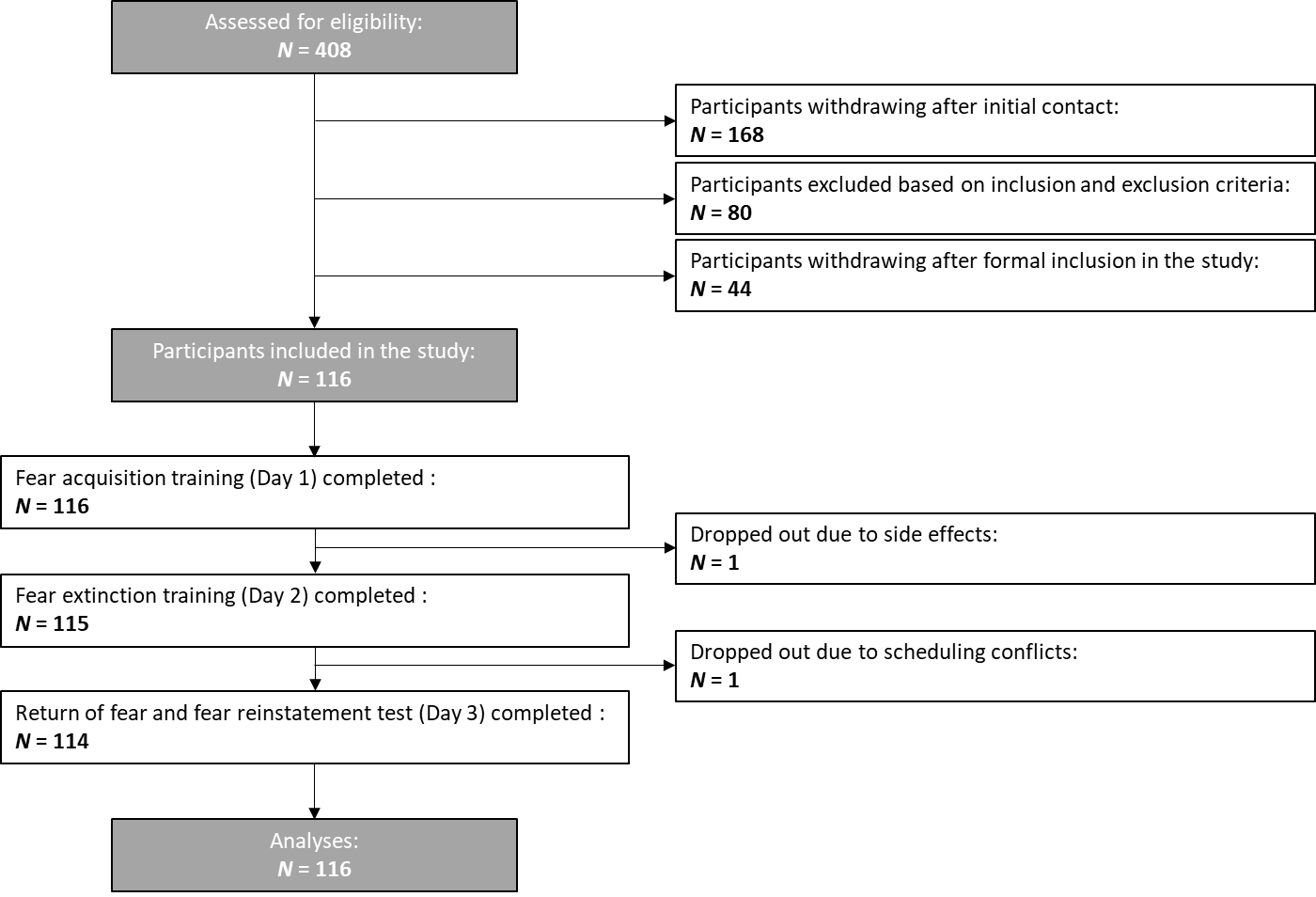


**Figure S2**

Depiction of saliva estradiol and progesterone concentrations on day 1, day 2, and day 3 broken up for all treatment groups.

**Estradiol** **Progesterone**

**Day 1: Fear acquisition training**


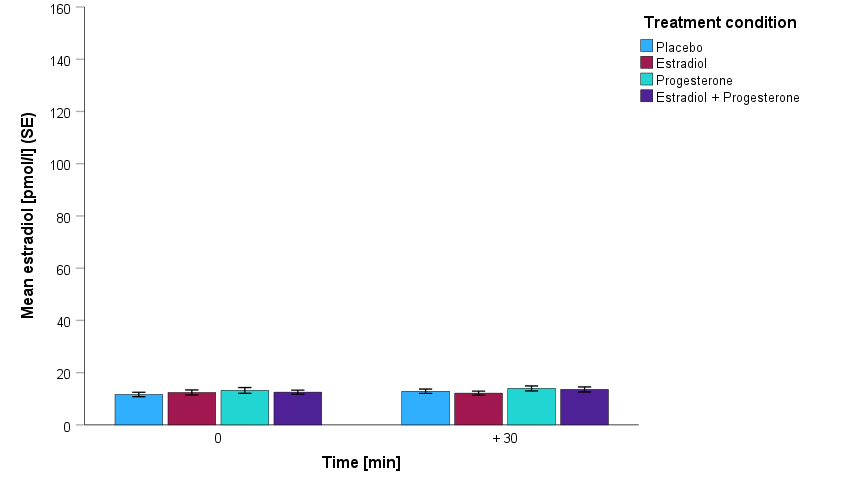

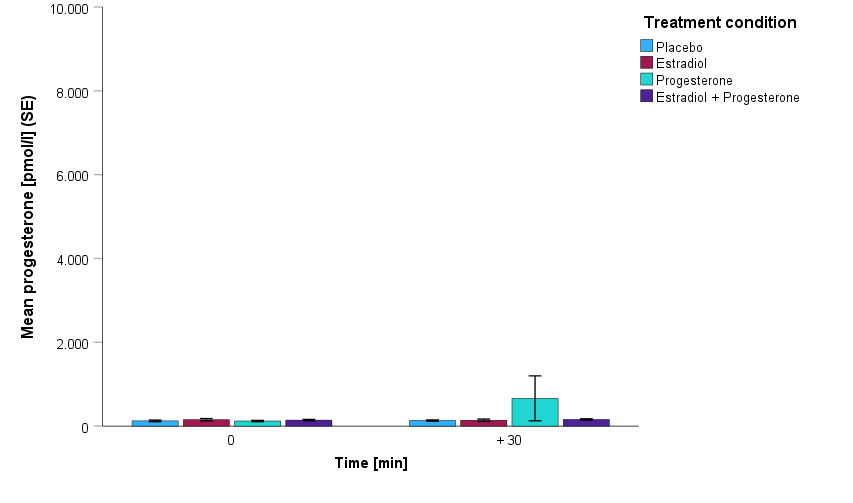


**Day 2: Fear extinction training**


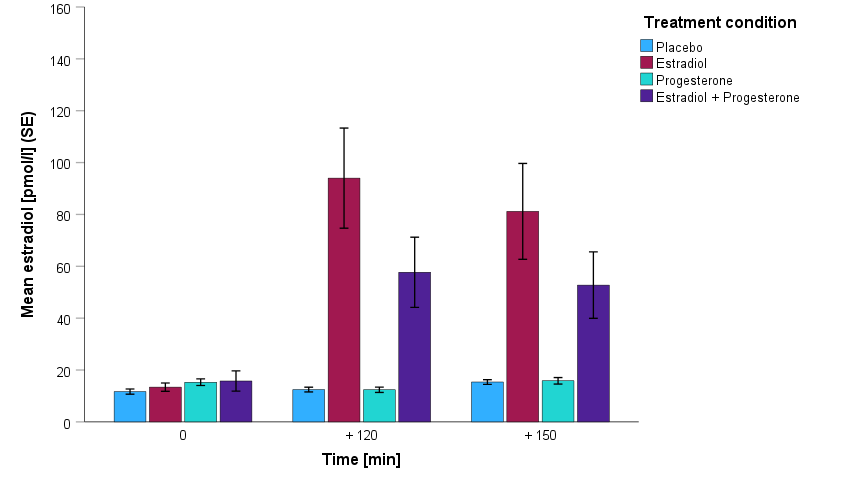

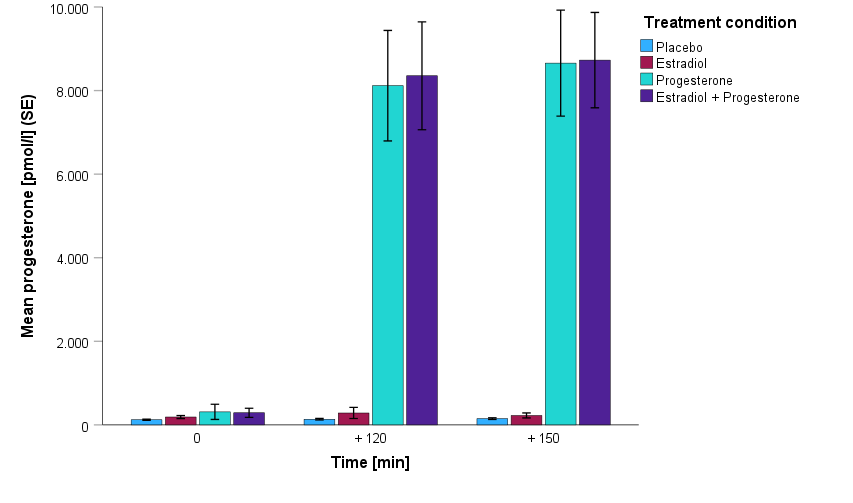


**Day 3: Return of fear and fear reinstatement test**


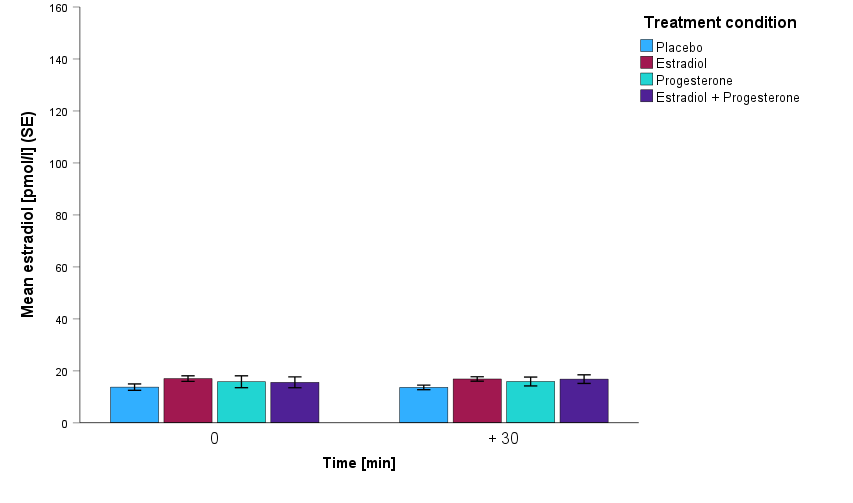

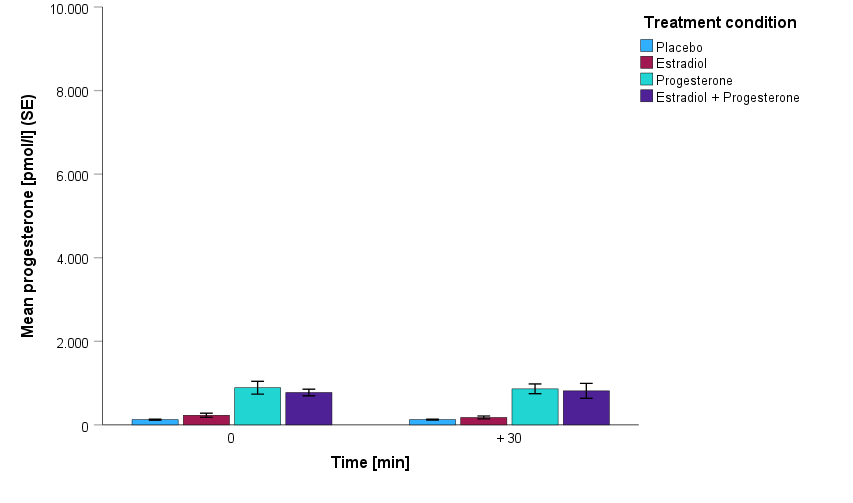


Manipulation check: depiction of saliva estradiol (lest side) and progesterone (right side) concentrations on day 1 and 3 before (0 min) and after testing (+30 min) and on day 2 before drug intake (0 min) as well as before (+120 min) and after fear extinction training (+150 min). A significant time by treatment interaction indicated significantly higher estradiol and progesterone concentrations after the respective drug administration (2 mg estradiol; 400 mg progesterone) on day 2.

**Figure S3**

Fear acquisition training (Day 1) broken up into the four treatment groups.

a) Placebo b) Progesterone


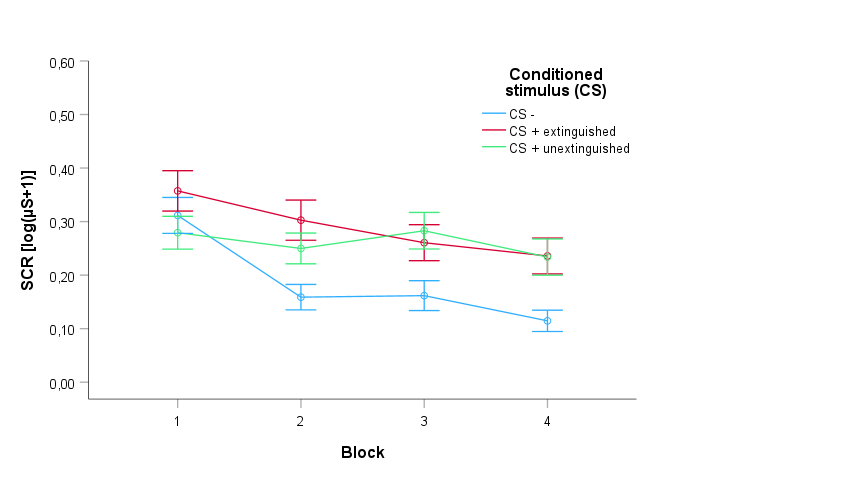

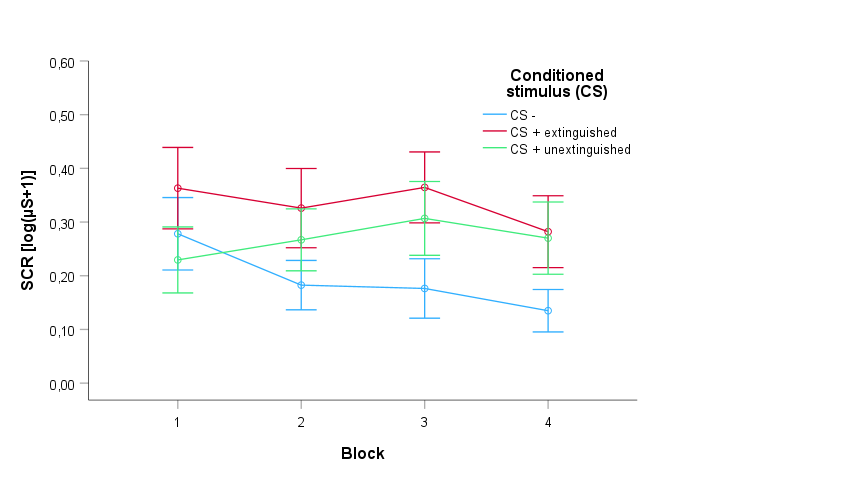


c) Estradiol d) Estradiol and progesterone


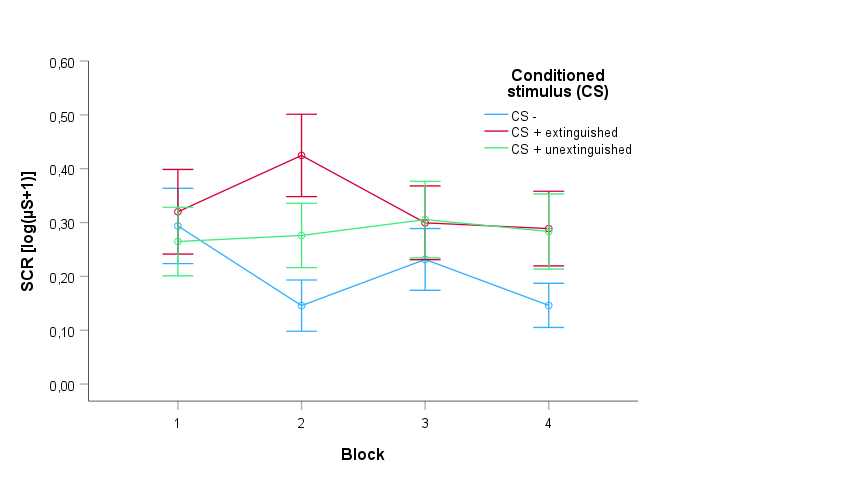

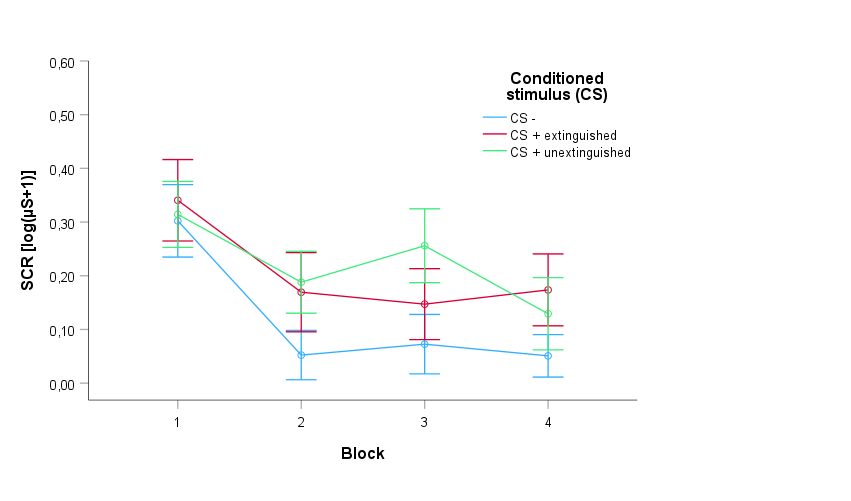


Depiction of the SCRs regarding the three conditioned stimuli (CS-, CS+E, CS+U) over the course of four blocks during fear acquisition training on day 1. Both CS+ were associated with significantly higher SCRs compared to the CS- in block 2, 3 and 4. There were no significant differences in SCRs between the two CS+.

**Table S1**

Additional analyses: Fear extinction training (Day 2).

Post-hoc *t*-test CS- vs. CS+E on day 2 (fear extinction training), separate for treatment groups and blocks, **bold** typeface indicates significant effects after Bonferroni-adjustment.

**Table S2**

Additional analyses: Return of fear and fear reinstatement test (Day 3).

**no Estradiol**

t

df

p

**Estradiol**

t

df

p

Block1

CS- / CS+E

-1,910

57

,061

CS- / CS+E

-4,760

55

**,000**

CS- / CS+U

-3,315

57

**,002**

CS- / CS+U

-4,905

55

**,000**

Block2

CS- / CS+E

-1,180

57

,243

CS- / CS+E

-,235

55

,815

CS- / CS+U

-2,321

57

,024

CS- / CS+U

,801

55

,427

Block3

CS- / CS+E

-2,219

57

,030

CS- / CS+E

-,806

55

,424

CS- / CS+U

-2,229

57

,030

CS- / CS+U

-,719

55

,475

Block4

CS- / CS+E

-,799

57

,428

CS- / CS+E

,744

55

,460

CS- / CS+U

,071

57

,944

CS- / CS+U

-,427

55

,671

Post-hoc *t*-test CS- vs. CS+E and CS- vs. CS+U on day 3 (return of fear test), separate for the estradiol vs. no estradiol treatment conditions, **bold** typeface indicates significant effects after Bonferroni-adjustment.

**Table S3**

Additional analyses: Fear extinction training (Day 2) as well as return of fear and fear reinstatement test (Day 3).

| **Day 2 - Fear extinction training** |  |  |  |  |
| --- | --- | --- | --- | --- |
| **CS-** |  |  |  |  |
|  | *df* | *F* | *p* | *partial eta²* |
| time | 1,000 | 19,073 | ,000 | ,147 |
| time * Estradiol | 1,000 | 3,895 | ,051 | ,034 |
| time * Progesterone | 1,000 | ,021 | ,884 | ,000 |
| time * Estradiol * Progesterone | 1,000 | 1,436 | ,233 | ,013 |
|  |  |  |  |  |
| **CS+E** |  |  |  |  |
| time | 2,668 | 32,848 | ,000 | ,228 |
| time * Estradiol | 2,668 | ,451 | ,503 | ,004 |
| time * Progesterone | 2,668 | ,100 | ,752 | ,001 |
| time * Estradiol * Progesterone | 2,668 | ,040 | ,842 | ,000 |
|  |  |  |  |  |
|  |  |  |  |  |
|  |  |  |  |  |
| **Day 3 - Return of fear test** |  |  |  |  |
| **CS-** |  |  |  |  |
|  | *df* | *F* | *p* | *partial eta²* |
| time | 2,861 | 5,600 | ,001 | ,048 |
| time * Estradiol | 2,861 | 2,124 | ,100 | ,019 |
| time * Progesterone | 2,861 | 1,899 | ,133 | ,017 |
| time * Estradiol * Progesterone | 2,861 | ,532 | ,652 | ,005 |
|  |  |  |  |  |
| **CS+E** |  |  |  |  |
| time | 2,668 | 25,767 | ,000 | ,190 |
| time * Estradiol | 2,668 | 1,772 | ,159 | ,016 |
| time * Progesterone | 2,668 | ,442 | ,700 | ,004 |
| time * Estradiol * Progesterone | 2,668 | ,033 | ,987 | ,000 |
|  |  |  |  |  |
| **CS+U** |  |  |  |  |
| time | 2,141 | 36,080 | ,000 | ,247 |
| time * Estradiol | 2,141 | 1,474 | ,230 | ,013 |
| time * Progesterone | 2,141 | ,773 | ,471 | ,007 |
| time * Estradiol * Progesterone | 2,141 | 2,787 | ,060 | ,025 |

rmAMOVA separate for CS-type: Fear extinction training and return of fear test.
